# Sequential learning of psychomotor and visuospatial skills for laparoscopic suturing and knot tying – study protocol for a randomized controlled trial “The shoebox study”

**DOI:** 10.1186/s13063-015-1145-8

**Published:** 2016-01-07

**Authors:** Jonathan D. Hendrie, Felix Nickel, Thomas Bruckner, Karl-Friedrich Kowalewski, Carly R. Garrow, Maisha Mantel, Philipp Romero, Beat P. Müller-Stich

**Affiliations:** Department of General, Visceral, and Transplantation Surgery, University of Heidelberg, Im Neuenheimer Feld 110, 69120 Heidelberg, Germany; Institute for Medical Biometry and Informatics, University of Heidelberg, Im Neuenheimer Feld 305, 69120 Heidelberg, Germany; Department of Pediatric Surgery, University of Heidelberg, Im Neuenheimer Feld 110, 69120 Heidelberg, Germany

**Keywords:** Laparoscopy, Training, Education, Minimally invasive surgery, Suturing, Knot tying

## Abstract

**Background:**

Laparoscopy training has become an integral part of surgical education. Suturing and knot tying is a basic, yet inherent part of many laparoscopic operations, and should be mastered prior to operating on patients. One common and standardized suturing technique is the C-loop technique. In the standard training setting, on a box trainer, the trainee learns the psychomotor movements of the task and the laparoscopic visuospatial orientation simultaneously. Learning the psychomotor and visuospatial skills separately and sequentially may offer a more time-efficient alternative to the current standard of training.

**Methods:**

This is a monocentric, two-arm randomized controlled trial. The participants are medical students in their clinical years (third to sixth year) at Heidelberg University who have not previously partaken in a laparoscopic training course lasting more than 2 hours. A total of 54 students are randomized into one of two arms in a 1:1 ratio to sequential learning (group 1) or control (group 2). Both groups receive a standardized introduction to the training center, laparoscopic instruments, and C-loop technique. Group 1 learn the C-loop using a transparent shoebox, thus only learning the psychomotor skills. Once they reach proficiency, they then perform the same knot tying procedure on a box trainer with standard laparoscopic view, where they combine their psychomotor skills with the visuospatial orientation inherent to laparoscopy. Group 2 learn the C-loop using solely a box trainer with standard laparoscopic view until they reach proficiency. Trainees work in pairs and time is recorded for each attempt. The primary outcome is mean total training time for each group. Secondary endpoints include procedural and knot quality subscore differences. Tertiary endpoints include studying the influence of gender and video game experience on performance.

**Discussion:**

This study addresses whether the learning of the psychomotor and visuospatial aspects of laparoscopic suturing and knot tying is optimal sequentially or simultaneously, by assessing total training time, procedural, and knot quality differences between the two groups. It will improve the efficiency of future laparoscopic suturing courses and may serve as an indicator for laparoscopic training in a broader context, i.e., not only for suturing and knot tying.

**Trial registration:**

This trial was registered on 12 August 2015 with the trial registration number DRKS00008668.

## Background

Minimally invasive surgery (MIS) involves learning a separate skill set from that of open surgery. Although some of the necessary qualities are the same, e.g., bimanual dexterity and steadiness, many of the basic skills must be learned for the first time or anew, e.g., the fulcrum effect, tissue grasping, needle manipulation, knot tying, etc. These basic psychomotor skills give surgeons the tools necessary to complete full operations; therefore, the learning of them has become one of the cornerstones of laparoscopic training curricula [1–3]. As many surgical operations defer to the laparoscopic approach, it has become imperative for surgical trainees to demonstrate proficiency in basic and procedural skills prior to operating on patients. Training modalities, e.g., virtual reality (VR) simulators, inanimate box trainers, and cadaveric organ trainers can be used as a safe, ethical, and effective means to do this [4–8].

Box trainers provide a realistic platform for the learning of laparoscopic skills with real instruments [9]. Intracorporeal suturing and knot tying [10] is an invaluable skill needed for minimally invasive operations [11, 12]. Suturing skills learned via a box trainer suture model have been shown to transfer to the operating room (OR) [13]. Training within the box trainer familiarizes trainees with the two major facets of laparoscopy: psychomotor control and visuospatial orientation [14–18].

Surgeons need to be familiar with depth perception and 2D–3D visuospatial understanding, since laparoscopy utilizes an indirect view. However, it remains to be determined if the learning of psychomotor and visuospatial skills simultaneously is optimal, as in a box trainer. Learning these two skills separately and sequentially may offer a more effective training alternative that reduces the learning or teaching time for trainees and mentors respectively. This would lower the workload of tutors and the learning curve of trainees, and save limited time and resources in a training center. Therefore, we aim to explore whether sequential learning with a transparent shoebox, where one learns the psychomotor movements, followed by training on a box trainer with the indirect laparoscopic view, expedites the learning of the surgical C-loop technique in comparison to training solely on a box trainer with the indirect laparoscopic view, where one learns the psychomotor and visuospatial skills simultaneously.

## Methods

### Objective

**Fig. 1:**
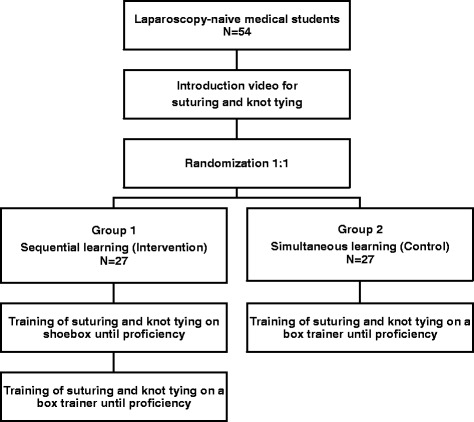
Study flowchart

The primary goal of this study is to identify if students in group 1, who learn the surgical C-loop technique using a transparent shoebox before using a box trainer with laparoscopic view, have a shorter learning curve than students in group 2, who learn the technique using solely a box trainer with laparoscopic view (Fig. 1). Secondary endpoints include number of attempts taken to gain proficiency, and examining procedural and knot quality subscore differences. In subgroup analyses we will separately explore possible gender differences and influence of previous video game experience on training time, number of attempts needed to reach proficiency and knot quality respectively.

### Study design

This is a registered prospective, single-center, two-arm, parallel group randomized controlled trial.

### Setting and participants

This study is conducted in the MIS training center of Heidelberg University Hospital’s Department of General, Visceral, and Transplantation Surgery. It offers a voluntary laparoscopic training course to medical students. Training tutors are specially trained medical students (*n* = 4) at Heidelberg University, non-blinded to the training groups, who receive a standardized rater training prior to the beginning of data collection.

### Inclusion and exclusion criteria

The inclusion criterion mandates that participants are medical students in their clinical years (third to sixth year) at Heidelberg University. Exclusion criteria include students who have previously participated in a laparoscopic training course of more than 2 hours duration or who had training in laparoscopic surgery in the OR.

### Video introduction to laparoscopic suturing and knot tying

All students receive a standardized video-based introduction to the suturing and knot tying technique at the start of the study. Students may refer to this video throughout the course of their participation.

### Introduction to the training modalities in the training center

Trained student tutors provide a standardized introduction for the box trainer, shoebox, and laparoscopic instruments, as well as instructions for their use. Students can, therefore, familiarize themselves with the training modalities, workspace, and terminology prior to the induction of their training.

### Randomization

Participants are randomly allocated to either the sequential learning group (group 1) or control group (group 2) in a 1:1 ratio. The randomization of subjects is performed by an employee otherwise not involved in the study using sealed envelopes labeled by block. Trainees are allocated to groups without stratification by gender or gaming experience. Although this study would benefit from a 1:1 ratio of men to women, this cannot be mandated since the data is collected through participation in a university elective course which is offered on a first-come-first-serve basis. Consequentially, we cannot explore outcome differences in heterogeneous or homogeneous groups, e.g., male-female, male-male, female-female, in a standardized manner; there may be an influencing factor that stems from the communication dynamic, previous experience of a partner, etc. We aim to explore these differences by comparing outcomes between pair group subgroup analysis following data acquisition. An employee of the Department of Medical Biometry at Heidelberg University, who is otherwise not involved with the training, tests, or data collection from the present study, assigned block randomization.

### Training curriculum

**Fig. 2:**
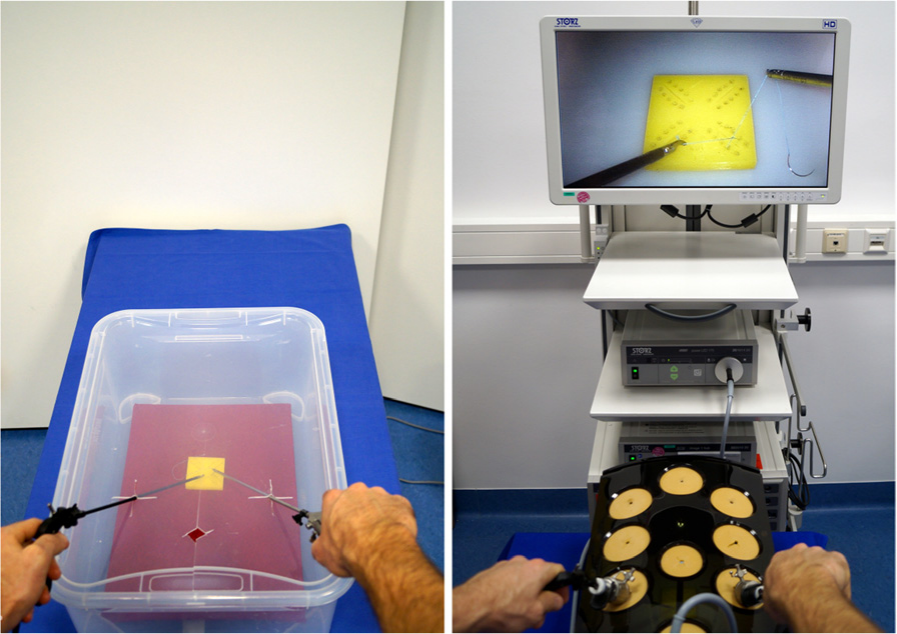
Transparent shoebox used by intervention group (left). Box trainer with laparoscopic view used by both groups (right)

The sequential learning group (group 1) learn the psychomotor aspects of laparoscopic suturing and knot tying first on the transparent shoebox without having to adjust to the visuospatial orientation inherent to the laparoscopic view of a box trainer. Once they reach the predefined proficiency criteria explained below (Tables 1, 2 and 3), these students then train on the box trainer with laparoscopic view until proficient (Fig. 2). The control group (group 2) learn the psychomotor skills and visuospatial aspects of laparoscopy simultaneously, as is traditionally done. They train using the box trainer with laparoscopic view until proficient in the predefined criteria. Specially trained peer tutors assist trainees during the course of the study and are available on demand in the training room for both groups; this has proven to be beneficial to trainee learning [19].

**Table 1:** Procedural proficiency checklist

Procedure assessment			Yes/No
Needle position 1	1	Held at one half to two thirds distance from the tip	
	2	Angle 90° ± 20°	
	3	Uses tissue or other instrument for stability	
	4	Attempts at positioning (≤3)	
Needle driving 1 (entry to incision)	5	Entry at 60° to 90° to the tissue plane	
	6	Driving with one movement	
	7	Single point of entry through the tissue	
	8	Removes the needle along its curve	
Needle position 2	9	Held at one half to two thirds distance from the tip	
	10	Angle 90° ± 20°	
	11	Uses tissue or other instrument for stability	
	12	Attempts at positioning (≤3)	
Needle driving 2 (incision to exit)	13	Driving with one movement	
	14	Removes the needle along its curve	
Pulling the suture	15	Needle on needle holder in view at all times	
	16	Uses the pulley concept	
Technique of knots	17	Correct C-loop (no S- or O-loops)	
	18	Smoothly executed throw, no fumbles	
	19	Correct inverse C-loop (no S- or O-loops)	
	20	Smoothly executed throw, no fumbles	
	21	Knot squared (capsized/reef/surgical)	
	22	Correct third C-loop (no S- or O-loops)	
	23	Smoothly executed throw, no fumbles	
Total points

**Table 2:** Knot quality checklist

Knot quality assessment	Available points
No visible gaps between stacked throws	1
Knot tight at base	1
Only edges are opposed (no extra tissue in knot, e.g., back wall)	1
Knot holds under tension	2
Maximum	5

**Table 3:** Competency checklist

Competency assessment	Goal	Yes/No
Time (min:sec)	≤01:15	
Procedure	≥18	
Knot quality	≥4	
Accuracy (mm)	≤2	
Competency (if all “Yes” above)

Following randomization, participants are put into training pairs, which is a common practice within surgical training [20, 21]. This allows the trainees to give each other real-time competency assessment and feedback, which is motivational [22, 23], in addition to saving time for the trainers, and space in the training center. We recently explored the difference between laparoscopic training alone and in pairs and found there to be no distinction in outcome between the two groups (protocol, own data, unpublished). It has been shown that students – whether alone or in a collaborative pair – can learn as much by observing their peers learn a task as they can by performing the task themselves [24, 25]. This is known as vicarious learning, which typically does not provide for communication between the student being tutored and the student watching. It should be made clear that students in our training environment can communicate directly with one another, which may prove even more useful. Based on these grounds, we believe that the pragmatic aspect of saving time and resources in a busy training center outweighs the potentially confounding effect of partner training.

**Fig. 3:**
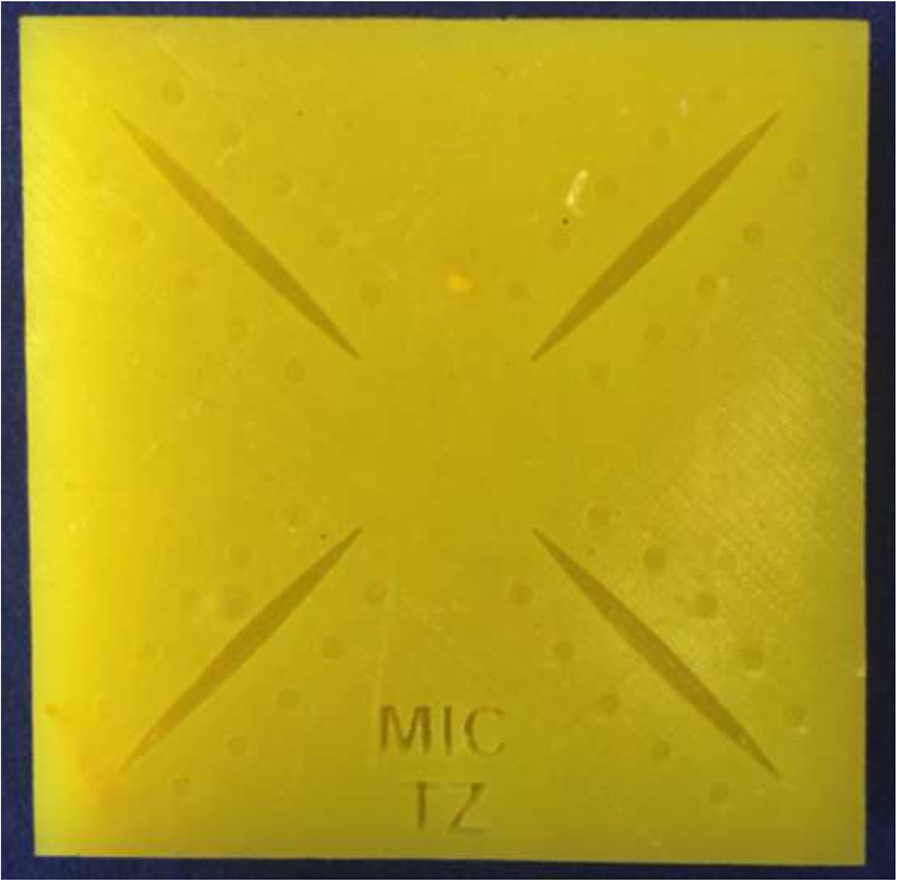
Standardized silicone suture pad

The student who is watching records the time taken for each attempt of the operating partner, starting from when the needle is grasped and ending once the final knot is tied. All attempts must be completed and the time per attempt must be recorded for each trainee. In the event of technical or instrument failure, e.g., the suture thread getting stuck in the grooves of the instrument, time is stopped and recorded, and “N/A” and a brief description of what happened are written next to the attempt. Rather than mass practice, we implement the more effective interval training [26–28]; trainees are required to switch positions at least every five completed knot attempts. Students train using two laparoscopic needle holders (KARL STORZ GmbH & Co. KG, Tuttlingen, Germany) and a standardized silicone suture pad with diagonal incisions and predefined suture entry and exit points (Fig. 3) (Big Bite Medical GmbH, Heidelberg). The suture material is a Polysorb 3-0 braided absorbable suture with a CV-23 taper ½ 17-mm needle (Covidien^TM^, Minneapolis, MN, USA).

Training pairs are required to rate each other throughout the training process until they reach the predefined competency levels for knot quality, suture position, and time. Procedural competency is assessed using a previously validated modified 23-point implementation checklist [14] (Table 1), originally published by Munz et al. [29].

Knot quality is scored using a 5-point scale designed by Muresan et al. [10], which assesses a knot’s throws, tightness, edge opposition, and ability to hold under tension (Table 2). Furthermore, accuracy is recorded by measuring the distance (mm) of the stitch from the edge of the entry and exit points of the standardized suture pad. Operative time is also recorded.

A trainee demonstrates competency for the C-loop upon finishing it in ≤01:15 (min:sec), attaining ≥18 points on the procedural implementation checklist, scoring ≥4 on the knot quality scale, and maintaining stitching ≤2 mm from the edge of the suture pad’s entry and exit points (Table 3). According to the available medical literature, experienced surgeons (expert level) reach these goals for the surgical C-loop [14]. Once a student has reached competency according to his or her training partner, a specially trained peer tutor is asked to provide expert assessment. If the participant then performs a C-loop, attaining the aforementioned competency criteria under supervision of the tutor, he or she is considered proficient in the technique; thus proficiency is achieved once a trainee completes the C-loop suturing technique on two consecutive occasions with the predefined competency criteria – once for the partner and once for the peer tutor assessment.

### Primary endpoint

The primary endpoint is total training time needed to reach proficiency in a predefined standardized suturing and knot tying technique.

### Secondary endpoints

Secondary endpoints include the number of attempts, procedure subscore differences, and knot quality subscore differences.

### Tertiary endpoints

Tertiary endpoints involve subanalyses of gender differences and the influence of video game experience. Surgery has traditionally been a male-dominated field and research has demonstrated that male medical students are faster at acquiring surgical skills and demonstrate superior visuospatial skills in comparison to their female peers [30–33]. We will explore this further by comparing male and female performance in both groups for total time taken and number of attempts. Furthermore, we will explore the influence of video game experience, in years, on total time taken and number of attempts, for both groups.

### Statistical analysis

The empirical distributions of all parameters of interest are described using mean and standard deviation in case of continuous data, with absolute and relative frequencies in case of categorical parameters. Possible difference in the primary outcome, time to reach proficiency, will be tested using a two-sided *t* test. All secondary parameters including subgroup analyses will be descriptively analyzed according to their underlying distribution, two-sided Mann-Whitney *U* tests in case of continuous parameters, and Chi-square test in case of categorical data. Graphical statistical methods will illustrate the findings, whenever appropriate.

### Sample size determination

Sample size calculation was done with respect to the primary endpoint. A previously evaluated pilot study showed the following data for total training time needed to reach proficiency: mean difference between group 1 and group 2 was 15 seconds; standard deviation in group 1 was 15.6 seconds, whereas it was 22.2 seconds in group 2. This difference can be detected with a two-sided significance level *α* = 0.05 and a power of 1 − *β* = 0.8, with a group size of 27 participants randomized to each group.

### Ethical and legal aspects

All data for the study is recorded anonymously, treated confidentially, and is evaluated by authorized staff for scientific purposes only. Participants’ names are kept separate from all study data and are not used for the study. Each participant is assigned a designated code that is used for the entire study documentation and data collection. This study is offered in addition to compulsory university courses. Participation in this study is voluntary and may be ended at any time. There are no foreseeable negative consequences related to participation. The participating staff of the Heidelberg MIS training center is experienced in the handling of training devices and in tutoring MIS. The benefits of training for students are numerous: stamina, concentration, and manual adroitness are enhanced and practiced, surgical interest may be sparked or strengthened, and students receive practical laparoscopy experience, which may be used during later work. In the event that a participant’s physical or mental health becomes jeopardized due to participation in the present study, the participant will be withdrawn immediately and excluded from the study. Written informed consent is obtained from each trainee. Ethical approval was obtained from the Ethics Committee of the Medical Faculty at Heidelberg University prior to the beginning of the study (Code S-334/2011). The Consolidated Standards of Reporting Trials (CONSORT) guidelines for randomized controlled trials and Standard Protocol Items: Recommendations for Interventional Trials (SPIRIT) guidelines for implementation of study protocols were followed [34, 35]. This trial was registered with the German Clinical Trials Register (DRKS) in Freiburg, Germany on 12 August 2015 with the trial registration number DRKS00008668.

## Discussion

This study aims to assess the differences in the learning of the psychomotor and visuospatial aspects of laparoscopic suturing and knot tying between those who learn them sequentially and those who learn them simultaneously. Trainees typically learn these aspects simultaneously, but no support was found necessitating this methodology. We expect that trainees who start on the shoebox will learn the C-loop much quicker than those who begin with the box trainer. However, it remains to be determined whether these skills quickly transfer to the box trainer with laparoscopic view. Nonetheless, it is important to ascertain which training method will be the most efficient for training centers. The potential for shorter learning curves and more effective use of resources, e.g., training center space, time, and trainers is rationale in itself to explore alternatives to current standards. The results of this study have the potential to shift the current paradigm for the training of laparoscopic suturing and knot tying or strengthen the present standard.

### Trial status

Recruitment started in April 2015 and is expected to finish by December 2015.

## Consent

Written informed consent was received from the participants for publication of this manuscript and accompanying images. A copy of the written consent is available for review by the editor-in-chief of this journal.

## Abbreviations

ICC: intraclass correlation coefficient
LC: laparoscopic cholecystectomy
MIS: minimally invasive surgery
OR: operating room
VR: virtual reality
